# Thymus Ontogeny in Frogs: T-Cell Renewal at Metamorphosis

**DOI:** 10.1155/1992/26251

**Published:** 1992

**Authors:** Louise A. Rollins-Smith, Patrick J. Blair, A. Tray Davis

**Affiliations:** Department of Pediatrics Division of Pediatric Immunology and Rheumatology Vanderbilt University School of Medicine, Nashville Tennessee 37232 USA

**Keywords:** Thymus ontogeny, *Xenopus laevis*, thyroid hormones, metamorphosis

## Abstract

Metamorphosis in amphibians presents a unique problem for the developing immune system. Because tadpoles are free-living, they need an immune system to protect against potential pathogens. However, at metamorphosis, they acquire a variety of new adultspecific molecules to which the tadpole immune system must become tolerant. We hypothesized that *Xenopus laevis* tadpoles may avoid potentially destructive antiself responses by largely discarding the larval immune system at metamorphosis and acquiring a new one. By implanting triploid (3N) thymuses into diploid (2N) hosts, we examined the influx and expansion of host T-cell precursors in the donor thymus of normally metamorphosing and metamorphosis-inhibited frogs. We observed that donor thymocytes are replaced by host-derived cells during metamorphosis, but inhibition of metamorphosis does not prevent this exchange of cells. The implanted thymuses export T cells to the spleen. This donor-derived pool of cells declines after metamorphosis in normally developing frogs but is retained to a greater extent if metamorphosis is inhibited. These studies confirm previous observations of a metamorphosis-associated wave of expansion of T cells and demonstrate that it is not dependent on the relatively high concentrations of thyroid hormones required for metamorphosis. Although some larval T cells persist through metamorphosis, others may be destroyed or the larval population is significantly diluted by the expanding adult population.


*Developmental Immunology*, 1992, Vol. 2, pp. 207–213
Reprints available directly from the publisher
Photocopying permitted by license only

© 1992 Harwood Academic Publishers GmbH
Printed in the United Kingdom

### Thymus Ontogeny in Frogs: T-Cell Renewal at Metamorphosis

LOUISE A. ROLLINS-SMITH,* PATRICK J. BLAIR, and A. TRAY DAVIS

*Department of Pediatrics, Division of Pediatric Immunology and Rheumatology, Vanderbilt University School of Medicine, Nashville, Tennessee 37232*

Metamorphosis in amphibians presents a unique problem for the developing immune system. Because tadpoles are free-living, they need an immune system to protect against potential pathogens. However, at metamorphosis, they acquire a variety of new adult-specific molecules to which the tadpole immune system must become tolerant. We hypothesized that *Xenopus laevis* tadpoles may avoid potentially destructive antiself responses by largely discarding the larval immune system at metamorphosis and acquiring a new one. By implanting triploid (3N) thymuses into diploid (2N) hosts, we examined the influx and expansion of host T-cell precursors in the donor thymus of normally metamorphosing and metamorphosis-inhibited frogs. We observed that donor thymocytes are replaced by host-derived cells during metamorphosis, but inhibition of metamorphosis does not prevent this exchange of cells. The implanted thymuses export T cells to the spleen. This donor-derived pool of cells declines after metamorphosis in normally developing frogs but is retained to a greater extent if metamorphosis is inhibited. These studies confirm previous observations of a metamorphosis-associated wave of expansion of T cells and demonstrate that it is not dependent on the relatively high concentrations of thyroid hormones required for metamorphosis. Although some larval T cells persist through metamorphosis, others may be destroyed or the larval population is significantly diluted by the expanding adult population.

KEYWORDS: Thymus ontogeny, *Xenopus laevis*, thyroid hormones, metamorphosis.

### INTRODUCTION

The immune system and immune response pattern of *Xenopus laevis* tadpoles are distinct from that of adults (reviewed in Flajnik et al., 1987; Du Pasquier et al., 1989). Tadpoles reject slowly or more frequently become tolerant of major histocompatibility complex (MHC)-disparate skin grafts than adults; and they generally do not reject skin grafts differing by minor histoincompatibilities whereas adults do (Chardonnens and Du Pasquier, 1973; DiMarzo and Cohen, 1982a, 1982b; Obara et al., 1983; Cohen et al., 1985; Rollins-Smith et al., 1988). The tadpole antibody response to given antigen is generally less heterogenous and antibodies have lower affinities than those of adults (Du Pasquier and Haimovich, 1976; Du Pasquier et al., 1979; Hsu and Du Pasquier, 1984). Classical class I MHC antigens are not expressed by tadpole cells until they near metamorphosis (Flajnik et al., 1986; Flajnik and Du Pasquier, 1988) and class II MHC antigens are expressed on different lymphocyte subpopulations in the tadpole and adult (Du Pasquier and Flajnik, 1990; Rollins-Smith and Blair, 1990a). All of these studies suggest a significant reorganization of the immune system at metamorphosis. Indeed there is evidence for a major loss of lymphocytes from thymus, spleen, and liver at metamorphosis (Du Pasquier and Weiss, 1973; Rollins-Smith et al., 1984; Cohen et al., 1985) that is exacerbated by thyroxine-driven precocious metamorphosis (Rollins-Smith et al., 1988). As has been shown for birds (Le Douarin and Jotereau, 1975; Jotereau and Le Douarin, 1982) and mammals (Jotereau et al., 1987), thymus ontogeny in amphibians is characterized by a succesion of waves of T-cell precursors moving into the thymus where they expand, differentiate, and leave as mature T cells (Turpen and Smith, 1989). In *X. laevis*, the third wave of detectable

*Corresponding author.

expansion occurs in association with metamorphosis (Turpen and Smith, 1989). Little is known about the intrathymic signals that result in attraction of T-cell precursors during receptive periods. We hypothesized that a thyroid hormone-dependent maturational event in the thymus might be responsible for the metamorphosis-associated period of attractiveness. By implanting triploid (3N) thymuses into age-matched diploid (2N) larval hosts that were allowed to metamorphose or were prevented from metamorphosing, we were able to test this hypothesis. The studies were designed to answer two questions. Is the influx and expansion of stem cells in the thymus at metamorphosis a thyroid hormone-dependent phenomenon? Do larval donor-derived T lymphocytes that colonize the spleen persist through metamorphosis? We observed that donor thymocytes are replaced by host-derived cells in about 60 days regardless of whether they undergo metamorphosis. Some donor-derived larval T cells persist in the spleen for at least 1 month after metamorphosis, but their numbers are low. Inhibition of metamorphosis results in the persistence of a greater number of these larval T cells.

### RESULTS

### Renewal of Thymocytes at Metamorphosis in Normally Developing Hosts

To study the influx and expansion of T-cell precursors in normally developing hosts, a 3N thymus was implanted into a number of age- and stage-matched 2N hosts at days 35–37 (stages 53–54). Hosts were sacrificed prior to, during, and after metamorphic climax. The analysis of one family of developing frogs is shown in Fig. 1. Prior to metamorphosis (days 58–64, stages 54–56). more than 70% of cells in the implanted thymus were of donor origin. At the beginning of metamorphic climax (days 71–77, stages 60–61), the average percent of host-derived cells had increased to 43.9. At the conclusion of metamorphosis (days 92–97, stages 65–66), virtually all donor thymocytes had been replaced by host cells (Fig. 1).

When implantation of the thymus was delayed until 63 days of age (stage 56), replacement of donor thymocytes occurred well after metamorphosis. In hosts examined at 85 days of age (stages 65–66), only 9.0% of the cells in the implanted thymus were host-derived. At 100 and 120 days of age, respectively, the implanted thymus contained about 18% and 87% of host-derived cells (Fig. 1). This experiment demonstrates a wave of immigration and expansion of T-cell precursors that is occurring over a fairly broad period of time from day 35 to greater than 63 days.

### Evidence That Immigration and Expansion of T-Cell Precursors Is Not Thyroid Hormone-Dependent

To examine the question of whether or not immigration of T-cell precursors is thyroid hormone-dependent, half of a group of 2N larvae implanted with a 3N thymus at days 37–42 were immersed in 0.1% sodium perchlorate to prevent further thyroid hormone-dependent development. If the attraction and expansion of T-cell precursors required a thyroid hormone-dependent maturational change in the thymus, we would expect to see few 2N host cells in the implanted thymuses of perchlorate-treated permanent larvae. Instead, we observed that host-derived cells migrated into and replaced the donor cells in these hosts in a pattern similar to that of untreated hosts. There were no significant

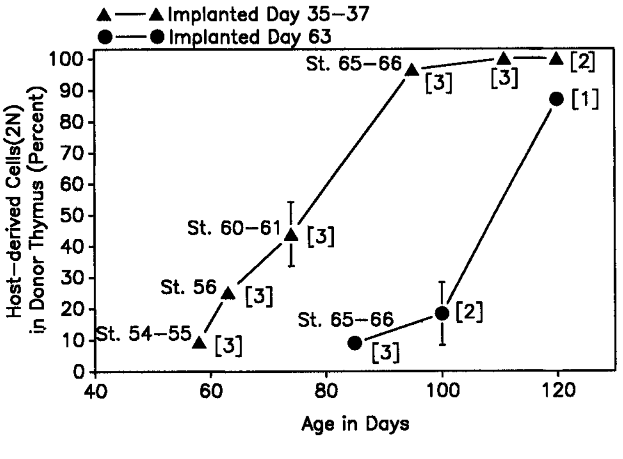

FIGURE 1 Percent (mean±SE) of host-derived (2N) cells in donor (3N) thymuses implanted at days 35–37 or day 63. The number of frogs sacrificed at each time point is shown in brackets. The developmental stage at sacrifice is shown next to each data point. This figure represents implantations done within a single family reared as described in Materials and Methods. Metamorphosis occurred between 80 and 100 days.

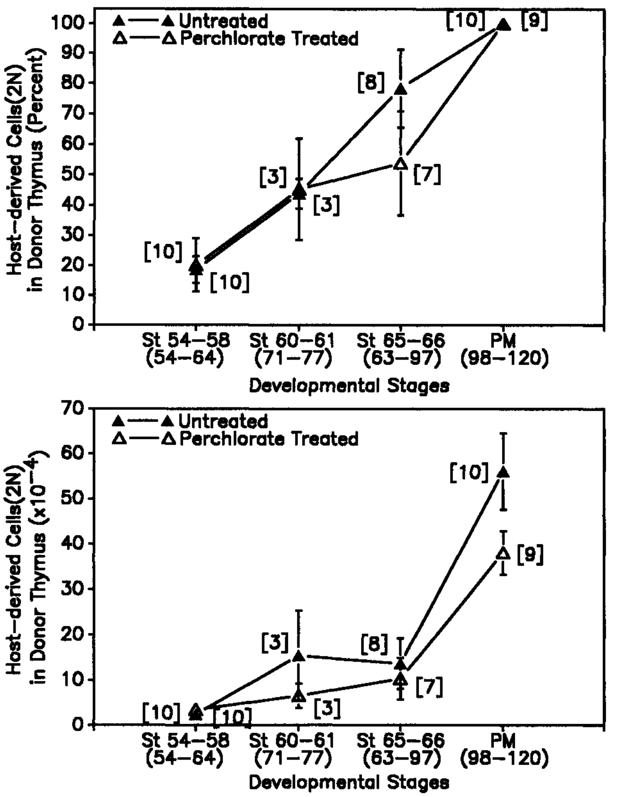

FIGURE 2 The numbers of host-derived (2N) cells in donor (3N) thymuses of untreated or perchlorate-treated hosts implanted at days 35–42 are shown as a percent (mean±SE) in the upper panel and as absolute numbers (mean±SE) in the lower panel. The number of frogs sacrificed at each stage is shown in brackets. The developmental stage and age in days postfertilization (in parentheses below stages) is shown for untreated frogs. Age-matched perchlorate-treated frogs were sacrificed on the same days as the untreated controls. They remained at larval stages 54–58 as judged by morphological characteristics. This figure represents the pooled results of implantations done within two separate families reared under similar conditions. Metamorphosis occurred between days 63 and 100.

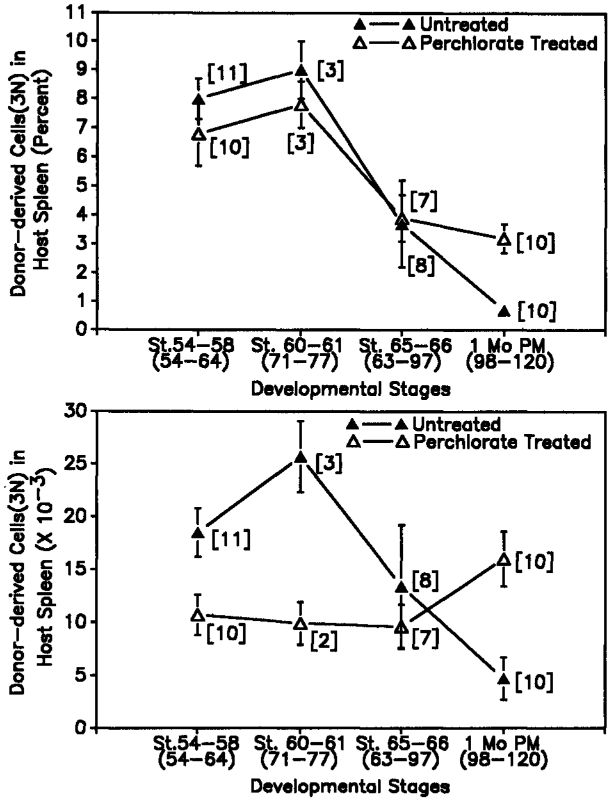

FIGURE 3 Immigration into host (2N) spleen by donor-derived (3N) T cells in untreated and perchlorate-treated hosts. Each data point represents the mean (±SE) number of donor-derived cells as a percent (upper panel) or as total recoverable cells (lower panel). The number of hosts sacrificed at each set of stages is shown in brackets. The developmental stage and age in days postfertilization (in parentheses below stages) are shown for untreated frogs. Age-matched perchlorate-treated frogs were sacrificed on the same days as the untreated controls. They remained at larval stages 54–58. This figure represents the pooled results of implantations done within two separate families reared under similar conditions. Metamorphosis occurred between days 63–100.

differences between perchlorate-treated and untreated frogs in the percent (Fig. 2, upper panel) or the absolute number (Fig. 2, lower panel) of host cells moving into the donor thymus. At 54–64 days, when untreated hosts were at larval stages 54–58, the implanted thymuses of both perchlorate-treated hosts and untreated hosts contained about 20% of host-derived cells. At 63–97 days, when untreated hosts were undergoing the climax of metamorphosis, implanted thymuses of both groups contained between 40 and 80% of host-derived cells. At 98–120 days of age, when untreated hosts were 1 month postmetamorphosis, the implanted thymuses of all perchlorate-treated and control hosts contained only host-derived lymphocytes. These experiments demonstrate that the influx and expansion of T-cell precursors at metamorphosis is not dependent on thyroid hormone.

### Persistence of Donor-Derived Larval (3N) T Cells in Metamorphosing and Metamorphosis-Inhibited Frogs

To examine the persistence of donor-derived larval (3N) T lymphocytes in the periphery of 2N hosts, we determined the percent of 3N cells in the host spleen of untreated and perchloratetreated hosts that had received a thymus implant at days 35–42. Host spleens of both groups contained about 7–8% donor-derived cells as early as 18–20 days postimplantation (54–64 days of age). At 1 month postmetamorphosis (57–85 days postimplantation), the spleens of untreated hosts retained only about 0.7% of donor thymus-derived cells, whereas those of perchlorate-treated hosts contained 3.2% (Fig. 3, upper panel). The difference in survival of donor T cells in perchlorate-treated hosts at 98–120 days of age is reflected not only in the percent of cells detectable, but also in the absolute numbers of recoverable cells (Fig. 3, lower panel, significantly different by Student's t-test, $p<0.01$). Thus, although the total spleen-cell population was significantly smaller in perchlorate-treated hosts at 90–120 days (data not shown), more cells were donor-derived.

### DISCUSSION

Our observation of a metamorphosis-associated wave of immigration and expansion of T-cell precursors confirms the previous observation of Turpen and Smith (1989) and extends the developmental time frame in which it occurs beyond day 63 (the latest day before metamorphosis at which thymus implants were done). This contrasts with the observations of Nagata and Kawahara (1982) of the replacement of donor thymocytes by host precursors in thymectomized adults 8–12 months of age. At 116 days postimplantation, less than 10% of the cells in implanted thymuses of these adult hosts were of host origin. Thus, rapid immigration of T-cell precursors appears to be associated with metamorphosis and the immediate postmetamorphic period but occurs much more slowly in older adults.

Although we cannot formally exclude the possibility that some cells from the host thymus migrated to the donor thymus, we believe it is unlikely because cells from the donor thymus were not detectable in the host thymus (data not shown). Thus, the most likely source of repopulation of the donor thymus was a peripheral stem-cell population.

Although perchlorate treatment prevented thyroid hormone-dependent morphological changes, it did not delay the replacement of 3N donor thymocytes by 2N host-derived cells. Perchlorate-treatment also does not prevent the expression of class I MHC antigens, which become detectable during metamorphic climax (stages 58–65) (Flajnik et al., 1986; Flajnik and Du Pasquier, 1988). Thus, immigration and expansion of T-cell precursors is coincident with class I MHC antigen expression. New T cells emerging from the thymus after metamorphic climax would therefore have antigen receptors selected in the presence of class I MHC antigens. This may help to explain why the skin graft rejection responses of postmetamorphic adults and age-matched goitrogen-blocked permanent larvae are better than those of younger larvae (DiMarzo and Cohen, 1982a, 1982b; Rollins-Smith et al., 1988).

Perchlorate-treatment does inhibit development of a set of adult-type T lymphocytes expressing class II MHC antigens and interferes with the expansion of thymocytes and splenocytes that occurs following metamorphosis (Rollins-Smith and Blair, 1990a). In the present study, host thymuses of perchlorate-treated frogs held significantly fewer thymocytes than those of untreated hosts at 1 month postmetamorphosis (data not shown). Thus, in perchlorate-treated permanent larvae, a new T-cell repertoire may be developing more slowly, and T cells emerging from the thymus have a larval rather than adult phenotype. Because perchlorate treatment did not prevent T-cell precursor immigration and expansion, the new T-cell repertoire may be building in the absence of a number of adult-specific molecules that appear only as a result of metamorphosis. If such permanent larvae are then released from the goitrogen block, they could theoretically develop destructive immune responses to some self-antigens.

Our studies of the persistence of donor-derived larval (3N) T cells in the spleen of 2N hosts show directly for the first time that some larval T cells persist for at least 1 month after metamorphosis. This was predicted from studies of T-dependent immunological memory that remains after metamorphosis (Du Pasquier and Haimovich, 1976; DiMarzo and Cohen, 1982b; Barlow and Cohen, 1983; Cohen et al., 1985; Manning and Al Johari, 1985) and the ability of frogs thymectomized just prior to metamorphosis to reject skin grafts 1–2 months postmetamorphosis (Barlow and Cohen, 1983). The experiments also suggest that metamorphosis results in the loss of some larval T cells and preventing metamorphosis may interfere with this loss of lymphocytes. Alternatively, the apparent loss of donor-derived larval cells may be due to their migration out of the spleen and dilution by the rapidly expanding adult lymphocyte population. At the present time, the available information does not allow us to distinguish between those two possibilities.

In summary, these studies show that there is a significant replacement of thymocytes at metamorphosis due to the entry of new peripheral precursors into the thymus. Although this immigration and expansion of new precursors are associated temporally with metamorphosis, they are independent of thyroid hormone-driven maturational changes. A cohort of larval T cells emerging from the thymus and migrating to the spleen during this period declines in numbers after metamorphosis.

Clearly, it is to the advantage of the tadpole to postpone development of most of its immune response capability until after metamorphosis when tolerance to the emerging adult-specific molecules can develop. Understanding precisely how the development of the immune system is coordinated with development of other systems is the goal of future studies.

### MATERIALS AND METHODS

### Frogs

MHC homozygous J-strain (Tochinai and Katagiri, 1975; DiMarzo and Cohen, 1982b) females were induced to ovulate by injection of human chorionic gonadotropin according to standard procedures (Rollins-Smith and Blair, 1990b). J-strain males were sacrificed to provide sperm for *in vitro* fertilization. Untreated and sodium perchlorate-treated frogs were reared as previously described (Rollins-Smith and Blair, 1990a, 1990b). Animal manipulations were performed in accordance with the USPHS guidelines as approved by the Vanderbilt University Animal Care Committee. Larval stages were determined according to the Normal Table of Nieuwkoop and Faber (1967).

### Production of Triploid (3N) Frogs

3N frogs were produced by the method of Kawahara (1978). *In vitro* fertilized eggs were immersed in cold (2–3 °C) 5% De Boer's solution (Katagiri, 1961) for 15 min at 15 min postferilization. Cold 5% De Boer's was then replaced by room temperature dechlorinatd tap water.

### Inhibition of Metamorphosis

Within 3 days after receiving a thymus implant (38–45 days of age), half of the hosts were placed in water containing 1 g/liter sodium perchlorate (Sigma). Water containing perchlorate was changed and animals were fed three times weekly.

### Thymus Implantations

3N larvae at 35–42 days (stages 53–54) or 63 days of age (stage 56) were sacrificed by immersion in 0.5% (w/v) tricaine methanesulfonate (ethyl m-aminobenzoate methanesulfonate; Crescent Research Chemicals, Phoenix, AZ) and blood was drawn from the heart with a capillary pipette previously wetted with heparin (Rollins-Smith and Blair, 1990b). Blood cells were diluted in L-15 medium (Sigma) containing 1% fetal calf serum, 100 IU/mL penicillin, and 100 $\mu$g/mL streptomycin for subsequent staining with propidium iodide (Krishan, 1975) and confirmation of ploidy by flow cytometry (Rollins-Smith and Blair, 1990b). Host 2N tadpoles were anesthetized by immersion in 0.01% tricaine methanesulfonate until they ceased to swim. 3N thymuses, cleared of adherent tissues, were inserted into a subdermal pocket located slightly anterior and medial to the eye.

### Preparation of Cells for Flow Cytometry

At various times after thymus implantation, hosts were killed by immersion in 0.5% tricaine methanesulfonate and the donor thymus, host thymuses, and host spleen removed and dissociated individually in 300 $\mu$L of L-15 medium in the well of a depression slide. Cells were collected, washed once, counted, and kept overnight at 4 °C in L-15 as previously described (Rollins-Smith and Blair, 1990b). Cells were stained with propidium iodide as previously described (Rollins-Smith and Blair, 1990b).

### Flow Cytometry

Cells were analyzed on an EPICS 753 flow cytometer (Coulter Electronics Inc., Hialeah, FL) as previously described (Rollins-Smith and Blair, 1990b). The integrated linear fluorescence signals, collected on a single cell population, were accumulated in 256 channel histograms and stored for subsequent analysis. Usually 10,000 nuclei were analyzed per sample. The percent of 2N and 3N cells present in each chimeric cell population was determined using the MODFIT analytical program (Verity Software House, Topsham, ME) (Bagwell, 1991).

### ACKNOWLEDGMENTS

We thank A. Lawton for reviewing the manuscript; W. Green and the flow cytometry laboratory of the Veterans Administration Medical Center of Nashville for assistance with flow cytometry; and M. Rardin for assistance in preparation of this manuscript. Supported by grants DCB-8710235 and DCB-9004666 from the National Science Foundation.

*(Received September 5, 1991)*

*(Accepted October 14, 1991)*

### REFERENCES

Bagwell C. (1991). Flow cytometry data analysis. *In:* Flow Cytometry: Principles and Clinical Applications, Bauer K., DuQue R., and Shankey T. V., Eds. (Baltimore: Williams and Wilkins), In Press.

Barlow E.H., and Cohen N. (1983). The thymus dependency of transplantation allotolerance in the metamorphosing frog, *Xenopus laevis*. Transplantation **35:** 612–619.

Chardonnens X., and Du Pasquier L. (1973). Induction of skin allograft tolerance during metamorphosis of the toad *Xenopus laevis:* A possible model for studying generation of self tolerance to histocompatibility antigens. Eur. J. Immunol. **3:** 569–573.

Cohen N., DiMarzo S., Rollins-Smith L., Barlow E., and Vanderschmidt-Parsons S. (1985). The ontogeny of allotolerlance and self-tolerance in larval *Xenopus laevis*. *In:* Metamorphosis, Balls M., and Bownes M. Eds. (Oxford: Oxford University Press), pp. 388–419.

DiMarzo S., and Cohen N. (1982a). An *in vivo* study of the ontogeny of alloreactivity in the frog, *Xenopus laevis*. Immunology **45:** 39–48.

DiMarzo S., and Cohen N. (1982b). Immunogenetic aspects of *in vivo* allotolerance induction during ontogeny of *Xenopus laevis*. Immunogenetics **16:** 103–116.

Du Pasquier L., Blomberg B., and Bernard C.C.A. (1979). Ontogeny of immunity in amphibians: Changes in antibody repertoires and appearance of adult major histocompatibility antigens in *Xenopus*. Eur. J. Immunol. **9:** 900–906.

Du Pasquier L., and Flajnik M.F. (1990). Expression of MHC class II antigen during *Xenopus* development. Dev. Immunol. **1:** 85–95.

Du Pasquier L., and Haimovich J. (1976). The antibody response during amphibian ontogeny. Immunogenetics **3:** 381–391.

Du Pasquier L., Schwager J., and Flajnik M.F. (1989). The immune system of *Xenopus*. Ann. Rev. Immunol. **7:** 251–275.

Du Pasquier L., and Weiss N. (1973). The thymus during the ontogeny of the toad *Xenopus laevis:* Growth, membrane-bound immunoglobulins and mixed lymphocyte reaction. Eur. J. Immunol. **3:** 773–777.

Flajnik, M.F., and Du Pasquier L. (1988). MHC class I antigens as surface markers of adult erythrocytes during the metamorphosis of *Xenopus*. Dev. Biol. **128:** 198–206.

Flajnik M.F., Hsu E., Kaufman J.F., and Du Pasquier L. (1987). Changes in the immune system during metamorphosis of *Xenopus*. Immunol. Today **8:** 58–64.

Flajnik M.F., Kaufman J.F., Hsu E., Manes M., Parisot R., and Du Pasquier L. (1986). Major histocompatibility complex-encoded class I molecules are absent in immunologically competent *Xenopus* before metamorphosis. J. Immunol. **137:** 3891–3899.

Hsu E., and Du Pasquier L. (1984). Ontogeny of the immune system in *Xenopus* II. Antibody repertoire differences between larvae and adults. Differentiation **28:** 116–122.

Jotereau F., Heuze F., Salomon-Vie V., and Gascan H. (1987). Cell kinetics in the fetal mouse thymus: Precursor cell input, proliferation, and emigration. J. Immunol. **138:** 1026–1030.

Jotereau F.V., and Le Douarin N.M. (1982). Demonstration of a cyclic renewal of the lymphocyte precursor cells in the quail thymus during embryonic and perinatal life. J. Immunol. **129:** 1869–1877.

Katagiri C. (1961). On the fertilizability of the frog egg I. J. Fac. Sci. Hokkaido Univ., Ser. 6. **14:** 607–613.

Kawahara H. (1978). Production of triploid and gynogenetic diploid *Xenopus* by cold treatment. Dev. Growth Differ. **20:** 227–236.

Krishan A. (1975). Rapid flow cytofluorometric analysis of mammalian cell cycle by propidium iodide staining. J. Cell Biol. **66:** 188–193.

Le Douarin N.M., and Jotereau F.V. (1975). Tracing of cells of the avian thymus through embryonic life in interspecific chimeras. J. Exp. Med. **142:** 17–40.

Manning M.J., and Al Johari G.M. (1985). Immunological memory and metamorphosis. *In:* Metamorphosis, Balls M., and Bownes M., Eds. (Oxford: Oxford University Press), pp. 420–433.

Nagata S., and Kawahara H. (1982). Thymocyte precursors in early-thymectomized *Xenopus:* Migration into and differentiation in allogeneic thymus grafts. Dev. Comp. Immunol. **6:** 509–518.

Nieuwkoop P.D., and Faber J. (1967). *Normal Table* of *Xenopus laevis* (Daudin) (Amsterdam: North Holl.)

Obara N., Kawahara H., and Katagiri C. (1983). Response to skin grafts exchanged among siblings of larval and adult gynogenetic diploids in *Xenopus laevis*. Transplantation **36:** 91–95.

Rollins-Smith L., and Blair P. (1990a). Expression of class II major histocompatibility complex antigens on adult T cells in *Xenopus* is metamorphosis-dependent. Dev. Immunol. **1:** 97–104.

Rollins-Smith L.A., and Blair P. (1990b). Contribution of ventral blood island mesoderm to hematopoiesis in postmetamorphic and metamorphosis-inhibited *Xenopus laevis*. Dev. Biol. **142:** 178–183.

Rollins-Smith L.A., Parsons S.C.V., and Cohen N. (1984). During frog ontogeny, PHA and Con A responsivenes of splenocytes precedes that of thymocytes. Immunology **52:** 491–500.

Rollins-Smith L.A., Parsons S.C.V., and Cohen N. (1988). Effects of thyroxine-driven precocious metamorphosis on maturation of adult-type allograft rejection responses in early thyroidectomized frogs. Differentiation **37:** 180–185.

Tochinai S., and Katagiri C. (1975). Complete abrogation of immune response to skin allografts and rabbit erythrocytes in the early thymectomized *Xenopus*. Dev. Growth Diff. **17:** 383–394.

Turpen J.B., and Smith P.B. (1989). Precursor immigration and thymocyte succession during larval development and metamorphosis in *Xenopus*. J. Immunol. **142:** 41–47.

